# Nutritional status and the influence of TV consumption on female body size ideals in populations recently exposed to the media

**DOI:** 10.1038/s41598-017-08653-z

**Published:** 2017-08-16

**Authors:** Jean-Luc Jucker, Tracey Thornborrow, Ulrik Beierholm, D. Michael Burt, Robert A. Barton, Elizabeth H. Evans, Mark A. Jamieson, Martin J. Tovée, Lynda G. Boothroyd

**Affiliations:** 1 Department of Psychology, Durham University, Durham, DH1 3LE UK; 2 Institute of Neuroscience, Newcastle University, Newcastle, NE1 7RU UK; 3 School of Psychology, University of Lincoln, Lincoln, LN6 7TS UK; 4 Department of Anthropology, Durham University, Durham, DH1 3LE UK; 5 Institute of Health and Society, Newcastle University, Newcastle, NE2 4AX UK; 6 School of Social Sciences, University of East London, London, E16 2RD UK; 7 Universidad de las Regiones Autónomas de la Costa Caribe Nicaragüense, Bluefields, Nicaragua

## Abstract

Television consumption influences perceptions of attractive female body size. However, cross-cultural research examining media influence on body ideals is typically confounded by differences in the availability of reliable and diverse foodstuffs. 112 participants were recruited from 3 Nicaraguan villages that differed in television consumption and nutritional status, such that the contribution of both factors could be revealed. Participants completed a female figure preference task, reported their television consumption, and responded to several measures assessing nutritional status. Communities with higher television consumption and/or higher nutritional status preferred thinner female bodies than communities with lower television consumption and/or lower nutritional status. Bayesian mixed models estimated the plausible range of effects for television consumption, nutritional status, and other relevant variables on individual preferences. The model explained all meaningful differences between our low-nutrition villages, and television consumption, after sex, was the most likely of these predictors to contribute to variation in preferences (probability mass >95% when modelling only variables with zero-order associations with preferences, but only 90% when modelling all possible predictors). In contrast, we found no likely link with nutritional status. We thus found evidence that where media access and nutritional status are confounded, media is the more likely predictor of body ideals.

## Introduction

Previous research has shown that the media, in particular television, can influence what people regard as an attractive female body, often with negative consequences for body satisfaction and self-esteem^1–5^. For example, a meta-analysis of 77 studies showed that the consumption of visual media, which predominantly feature unusually slim models, is related to a drive for thinness and body image concerns in White women^6^. Cross-cultural research has also shown than Non-Western samples with low access to the media tend to prefer larger female bodies than samples in the West^7–11^. It has also been suggested that the introduction of television in previously media-naive populations may decrease female body size preference in both men and women, and predicts dieting in women^12–14^.

Although previous research has provided evidence that the media can impact female body size ideals (including in Non-Western samples), it has not fully controlled for the potential crucial confounds related to nutritional status and food insecurity. In Non-Western samples, heavier bodies may be preferred not because of low access to the media, but because higher adiposity in women may be used as an index of good health, fertility, and adaptive value during periods of food scarcity^9^ or when the environment is less secure^15^. For example, research has shown that plump women are preferred in societies with limited access to food supplies^7, 10^, and that indigenous Nicaraguan women are encouraged to marry men who are good hunters, that is, good food suppliers for them and their offspring^16^. Furthermore, research in the West has shown that men who are about to have a meal prefer heavier women than men who have just eaten^17, 18^.

A recent study with a similar Nicaraguan sample attempted to control for current hunger by asking participants how long it had been since they had last eaten^14^. While this study found that television consumption remained the dominant predictor of preferences, the hunger data do not tap into the kind of long term nutritional stress which would have produced the adaptations hypothesised by Swami and colleagues^19^. Furthermore, Boothroyd *et al*.^14^ did not assess participants’ actual Body Mass Index (BMI), and utilised a diverse sample of participants in terms of ethnicity (Garifuna, Mestizo, and Miskitu) and acculturation (rural and urban dwellers). Finally, multicollinearity in the data prevented analyses which compared individual and location level effects on preferences. As such, not only did Boothroyd *et al*.’s study not assess long term nutritional stress, but it could not rule out the possibility that the relationship between television consumption and body size ideals or dieting may be mediated by other confounding variables.

The current study is the first to investigate the effect of media consumption on female body weight ideals while incorporating a comprehensive assessment of nutritional factors such as food insecurity, diet quality, current hunger, and participants’ actual BMI. We drew from the same region as Boothroyd *et al*.^14^ and selected three indigenous communities located around the Pearl Lagoon basin in Eastern Nicaragua. These three communities (hereafter Village A, Village B, and Village C) are predominantly of the same ethnic group (Garifuna) and share very similar cultural and environmental constraints with two important exceptions: Village A and Village B have access to television (since the year 2006 and 2009, respectively) whereas Village C has not, and Village A has better food supplies than both Village B and Village C. In other words, the communities selected represented three levels or combinations of television consumption and nutritional status: Village A had high TV access with high nutritional status, Village B had high TV access with low nutritional status, and Village C had low TV access with low nutritional status (Table 1).

**Table 1:** Study design.

	Nutritional status	
	High	Low
**TV access**		
High	Village A	Village B
Low	n/a	Village C

Our design allowed us to test three hypotheses. First, if female body ideals are constrained by nutritional factors alone, we would expect communities with low nutritional status to prefer heavier bodies irrespective of whether or not they have access to television. Second, if body ideals are constrained by television consumption alone, we would expect communities with television access to prefer thinner bodies irrespective of nutritional status. Third, we may also observe additive effects, such that a community with television access and low nutritional status would prefer heavier bodies than a community without television access and low nutritional status, but *not* than a community with television access and high nutritional status.

To test these hypotheses, we first assessed whether the three communities selected actually represented differing levels of television exposure and nutritional status. When this was confirmed, we ran comparisons between communities in order to identify any differences in female body size preferences. Finally we ran Bayesian regression analyses to determine whether the differences found between communities were better accounted for in terms of television consumption, nutritional status, or both. We also measured other important confounding variables of body ideals such as acculturation and socio-economic status.

## Method

### Study site

The study was conducted in the Pearl Lagon Basin of Eastern Nicaragua, a remote coastal lagoon that is home to twelve communities (collectively known as La Cuenca in Spanish) of predominantly indigenous Miskitu, Garifuna, and Creole people. These communities share many environmental and cultural constraints^20^, but differ in terms of our main variables of interest, therefore providing ideal conditions in which to conduct a naturalistic experiment. Out of the twelve villages, we were able to identify three ethnically-matched communities that differed both in terms of TV access and nutritional status, but were similar in almost every other regard: specifically three Garifuna, Creole-English speaking communities located within an eight-mile radius around the lagoon. Village B and C are small farming and fishing villages with a population of 52 and 38 adults, respectively. Village A, a larger community (approximately 700–750 adults; sex ratio: 1.07)^21^, has an economy also based on fishing and farming but with a greater degree of additional cash employment which facilitates more regular access to bought foods. The larger size of Village A also means that there are small shops selling food in the village, whereas villagers in our other locations have to travel by boat to other villages to buy additional foods.

Conversely, Villages A and B had access to grid electricity and satellite television, as well as DVD players and DVDs, whereas Village C had no access to electricity nor television at the time of data collection. In all three villages, and indeed in the region as a whole, magazines were not available. Furthermore, at the time of data collection, there was extremely limited access to the internet in our study site. Participants who had access to satellite TV reported watching a wide range of content (which was confirmed by participant observation), including programmes featuring women and actresses representing the thin ideal, such as *telenovelas* (Mexican and Latin American soaps), international news, Hollywood films and series, and North American documentaries. Participants were also exposed to advertisements while watching these programmes.

Thus our participants shared the same culture, social organisation, economic system, religious traditions, and food culture, but Village A had easier and more reliable access to a greater variety of bought foods than Villages B and C, while Village C had dramatically less access to visual media than Villages A and B.

### Participants

One hundred and twelve participants were recruited in Village A (*n* = 42), Village B (*n* = 40), and Village C (*n* = 30). As Village B and Village C are very small communities, our sampling rule was simply to test every available adult in these communities, which we did. In Village A, we used opportunity sampling and our rule was to test at least as many participants as in Village C, but not significantly more than in Village B, so that the three samples would have a similar size (note, these sample sizes give power of over 0.95 at alpha 0.05 to detect a pairwise difference of the same magnitude as seen in two villages in the region in our previous study^14^). The participants’ mean age was 31 years old (*SD* = 13.26; range: 15–77), and 46% (*n* = 51) of them were women; 76% (*n* = 84) of the participants identified as Garifuna or mixed Garifuna (statistics are presented separately for each village in Table 2).

**Table 2:** Means and standard deviations of the main variables of the study. Age range for Village A, B and C was 17-60, 15–74, and 16–77, respectively.

	All	Village A	Village B	Village C
Valid N	110	42	39	29
% female	45	48	44	41
% Garifuna	76	95	55	79
Acculturation	11.72 (1.81)	11.77 (1.94)	12.12 (2.16)	11.10 (0.49)
Age (years)	30.91 (13.11)	27.38 (9.68)	34.58 (14.47)	31.10 (14.51)
BMI	25.74 (6.28)	26.03 (7.53)	26.63 (5.63)	24.05 (4.78)
Diet quality	68.22 (13.24)	75.07 (12.84)	64.44 (10.05)	63.39 (13.78)
Earnings ($)	1,296 (1,259)	1,594 (1,272)	1,473 (1,401)	710 (806)
Economic Score	13.49 (5.66)	17.28 (4.88)	12.51 (4.59)	9.31 (4.52)
Education	8.35 (3.37)	9.59 (2.55)	8.28 (3.04)	6.65 (4.12)
Food insecurity	3.37 (1.59)	2.48 (1.53)	4.01 (1.56)	3.79 (1.11)
Hunger	4.61 (0.69)	4.90 (0.29)	4.35 (0.81)	4.55 (0.78)
Peak BMI preference	26.88 (3.90)	25.15 (3.11)	27.03 (4.15)	29.19 (3.42)
Size of last meal	1.48 (0.57)	1.85 (0.45)	1.58 (0.59)	1.48 (0.57)
TV consumption (hrs/week)	11.14 (8.18)	15.41 (7.46)	12.15 (7.29)	3.61 (4.42)
Time since last meal (hrs)	3.89 (3.33)	3.55 (2.87)	4.36 (3.65)	3.73 (3.53)
WHR	0.86 (0.07)	0.86 (0.08)	0.86 (0.06)	0.85 (0.05)

### Materials and measures

#### Nutrition

Participants’ nutritional status was assessed using the following measures. First, participants reported their level of hunger at the time of taking the study on a scale ranging from 1 (*famished, starving*) to 10 (*bursting, painfully full*). They also reported how long ago they had eaten (e.g., 3 hours and 15 minutes ago), and the size of that meal (*snack, medium meal, large meal*). On average, the participants reported a level of hunger of 4.61 (*SD* = 0.69, range: 3–6), they had taken their last meal 3.86 hours before taking part in the study (*SD* = 3.31; range: 0.25–15), and most of them had eaten a large meal (*n* = 78; 70%).

Second, participants reported how many times they consume each of 21 items in a typical week (7 days). These 21 items were the most common foods and beverages available in our study site: alcohol, beans, biscuits or crisps, bread or cake, breadkind (e.g., cassava, plantain), cheese, coffee or tea with sugar, deep fried foods, eggs, fish or seafood, fizzy soft drinks, fowl meat, fruits, pasta, powdered milk, processed meats, red meat (e.g., turtle, pork, beef), rice, squash or home-made lemonade, tobacco, and vegetables. Using a similar method as Clausen and colleagues^22^, the data collected were summed to obtain a diet quality score for each participant (i.e., the sum of how many times each participant consumed the 21 items in a week), such that a high diet quality score indicated a high quantity and variety of foods consumed. The average diet quality score was 68.32 (*SD* = 13.22; range: 42.5–99.0), out of a theoretical maximum of 147. Importantly, these data were used in cluster analyses to determine whether the participants’ diet differed by location in terms of nutritional value and not just quantity of food eaten (see Results section).

Third, participants were asked a series of questions assessing their food insecurity or seasonal risk of food scarcity. These questions reflected diverse indicators of food insecurity while taking into account the specificities of our study site. For example, participants were asked whether they had enough food on a typical day, whether they experienced periods of starvation in the year, and whether they considered that their community had better or poorer access to both quantity and variety of foods than surrounding communities (for the complete list of questions, see Supplementary Methods). Answers were summed to obtain a food insecurity score for each participant, with a high score indicating high food insecurity. The average food insecurity score was 3.37 (*SD* = 1.59; range: 0–8).

Finally, anthropometrics were measured to compute the Body Mass Index (BMI) and Waist to Hip Ratio (WHR) of each participant. The average BMI was 25.74 (*SD* = 6.28; range: 18.72–49.22) and the average WHR was 0.86 (*SD* = 0.07; range: 0.75–1.19).

#### Socio-economic status

Participants provided demographics and socio-economic status data. The average number of years of education by participant was 8.25 (*SD* = 3.45; range: 0–16), and their average annual income was equivalent to 1,284 US Dollars (*SD* = 1,257; range: 0–6,923) in local currency. As the economy of the Pearl Lagoon Basin is only partly based on cash^23^, we also administered a questionnaire assessing participants’ possessions and means of production, including dwellings, canoes and boats, fishing material, land, livestock, furniture, home appliances, etc. The data collected were summed to obtain an economic score by participant, with a high score indicating a high number of possessions and means of production. The average economic score was 13.44 (*SD* = 5.66; range: 1–27), out of a possible total of 33. Participants also completed an adapted version of the Suinn-Lew Self-Identity Acculturation Scale^24, 25^ for Hispanics^26^. This scale assesses the frequency with which participants speak, think, or socialise using the relevant ‘acculturated’ language (in this case, Spanish and US English) as opposed to using the ‘indigenous’ language (in this case, Creole English).

#### TV consumption

Participants reported whether they had access to a television (*in my house, in a neighbour’s house I visit, in a neighbour’s house I don’t visit, no TV in the village*), what type of television they had access to (*satellite TV vs. DVD player only*), and how many hours they had watched it in the last 7 days. Eighty-eight percent (n = 99) of the participants had a television in their own house or in a neighbour’s house they visit, and 69% (n = 78) had access to satellite television. This confirmed that approximately two thirds of our total sample were regularly exposed to a range of televisual programmes, including foreign programmes via satellite. Weekly television consumption was therefore used as our main measure of television consumption. On average, the participants watched television for a total of 11.17 hours in the 7 days preceding the experiment (SD = 8.15; range: 0–31.5).

#### Female figure preference task

Participants rated a set of photographs of women for attractiveness. This set has been used in previous published research^27^ and consists of 50 colour photographs of White women of known BMI in front view, at a standard distance and lighting conditions with their faces blurred and all wearing the same outfit (grey leotard and tights), and with ten bodies representing each of the five following BMI categories: <15 kg/m^2^; 15–19 kg/m^2^; 20–24 kg/m^2^; 25–30 kg/m^2^; and >30 kg/m^2^. Participants rated each body for how “attractive or good-looking” they thought they were, on a scale ranging from 1 (*very unattractive* or, in Creole English, *very bad body*) to 5 (*very attractive* or, in Creole English, *very good body*). The bodies were presented one-by-one on a laptop computer in an order that was randomised for each participant. Following Tovée *et al*.^18^, the participants’ ratings were used to compute the peak BMI preference of each participant by fitting a cubic regression function onto their preference ratings and the BMI of each body rated.

### Procedure

Participants were tested individually in a quiet room with a table. As most participants were not familiar with structured interviews and computer-based tasks, every effort was made to make them feel at ease, and their answers were entered on a laptop by the experimenter. It was explained that participation was voluntary, that they could stop the interview at any time, and that their individual answers would remain anonymous. The participants then completed the female figure preference task. Before rating the bodies, the participants were asked to write down the anchors and labels of the scale and to read them aloud; the rating task did not begin until the experimenter was convinced that the participant understood how to use the scale. The participants were then administered the questionnaires (demographics, acculturation, diet, etc.) orally. Finally, participants’ height, weight, chest, waist, and hips were measured using an electronic scale and tape measure; they were given the opportunity to take their measurements themselves (with guidance), and anthropometrics for women were collected by a female field assistant. All participants were interviewed in Creole English, and a typical session lasted 45–60 minutes. Each participant received the equivalent of 4 US Dollars in local currency for their time, even if they did not complete the full task. The methods and protocol used in this study were approved by the Durham Psychology Department Ethics Committee (ref 13/15). All methods were carried out in accordance with the relevant guidelines and regulations, and informed consent was obtained from all participants and/or their legal guardian/s.

### Data Availability

The datasets generated during and/or analysed during the current study are available from the corresponding author on reasonable request.

## Results

### Comparisons between samples

A series of ANOVAs and Tukey post hoc comparisons were used to investigate differences between locations on the control variables (means and standard deviations are shown in Table 2; the data of one participant who did not complete the task in full and of another participant who did not produce a viable peak BMI preference function were discarded from analyses).

There were no significant differences between locations in terms of acculturation (*F*_2, 104_ = 2.68, *p* = 0.073), BMI (*F*_2, 103_ = 1.41, *p* = 0.247), and WHR (*F*_2, 103_ = 0.02, *p* > 0.250). Residents of Village B were older than those of Village A (*F*_2, 107_ = 3.17, *p* = 0.046; post hoc *p* = 0.035), but not Village C (post hoc *p* > 0.250). Residents of both Village A and Village B earned more money in the previous year than residents of Village C (*F*_2, 95_ = 4.64, *p* = 0.012; post hoc *p*s < 0.036), but did not differ from each other (post hoc *p* > 0.250). Further, residents of Village A had a higher economic score than residents of Village B (*F*_2, 107_ = 26.12, *p* < 0.001; post hoc *p* < 0.001), who in turn had a higher economic score than residents of Village C (post hoc *p* = 0.017). Residents of Village A were also the most educated, but differed significantly only from residents of Village C (*F*_2, 107_ = 7.25, *p* < 0.001; post hoc *p* < 0.001), who did not differ from residents of Village B (post hoc *p* = 0.100). Finally, there were two overall sex differences such that women had a higher BMI (mean difference = 5.49, *t*_104_ = 4.41, *p* < 0.001), and a higher WHR (mean difference = 0.04, *t*_104_ = 3.45, *p* < 0.001) than men (the anthropometrics of three pregnant women were not included in the analyses, and there was no age difference between men and women; this unusual result may be explained by gender roles in our study site, where women tend to be more sedentary than men). There was however no interaction between sex and location for any variable (*F*s < 1.52, *p*s > 0.223).

#### TV consumption and nutrition

Further comparisons revealed that residents of Village C consumed less TV than residents of both Village B (*F*_2, 107_ = 27.02, *p* < 0.001; post hoc *p* < 0.001) and Village A (post hoc *p* < 0.001), who did not significantly differ from each other (post hoc *p* = 0.079). Further, residents of Village A had a higher diet quality (*F*_2, 107_ = 10.75, *p* < 0.001) and lower food insecurity (*F*_2, 107_ = 12.84, *p* < 0.001) than residents of both Village B and Village C (post hoc *p*s < 0.001), who did not differ from each other (post hoc *p*s > 0.250). Residents of Village A also reported a lower level of hunger than residents of Village B (*F*_2, 107_ = 7.24, *p* < 0.001; post hoc *p* < 0.001), and had a larger last meal than residents of Village C (*F*_2, 107_ = 5.25, *p* = 0.007; post hoc *p* = 0.008). Village B and Village C did not differ in terms or hunger (post hoc *p* > 0.250) or last meal size (post hoc *p* > 0.250), and time since last meal did not differ between any of the locations (*F*_2, 107_ = 0.63, *p* > 0.250).

Although these results confirmed that the three locations represented the three levels of TV consumption and nutrition (high TV and high nutritional status, high TV and low nutritional status, and low TV and low nutritional status) needed to test our hypothesis, cluster analysis was used to better assess the qualitative differences in diet between locations. When all participants and 19 items (alcohol and tobacco were not included) from the diet questionnaire were used, a two-step cluster analysis automatically classified the participants in two groups. Cluster 1 had 50 cases (45.5% of the participants), and Cluster 2 had 60 cases (54.5%); the ratio of sizes was 1.20 and the measure of cohesion and separation was qualified as ‘fair’. As one can see in Supplementary Table S1, participants in Cluster 1 had a richer (especially in proteins) and more varied diet than participants in Cluster 2. For example, participants in Cluster 1 consumed weekly at least twice as much fowl meat and red meat, bread, cheese, and vegetables, than participants in Cluster 2. Participants in Cluster 1 also consumed more beans, fruits, cooking oil, and processed foods, than participants in Cluster 2.

A chi-square test was used to determine if participants’ cluster membership was related to location, and found this to be the case (χ² = 25.913, df = 2, *p* < 0.001), such that residents of Village A were significantly more likely to belong to Cluster 1 than residents of both Village B (χ² = 20.698, df = 1, *p* < 0.001) and Village C (χ² = 16.475, df = 1, *p* < 0.001), who were significantly more likely to belong to Cluster 2 and who did not differ from each other (χ² = 0.032, df = 1, *p* > 0.250). This confirmed that the participants’ diet differed between communities, and in particular that the two communities with television access (Village A and Village B) represented the two levels of nutritional status needed to test our hypotheses.

#### Peak BMI preference

**Figure 1:**
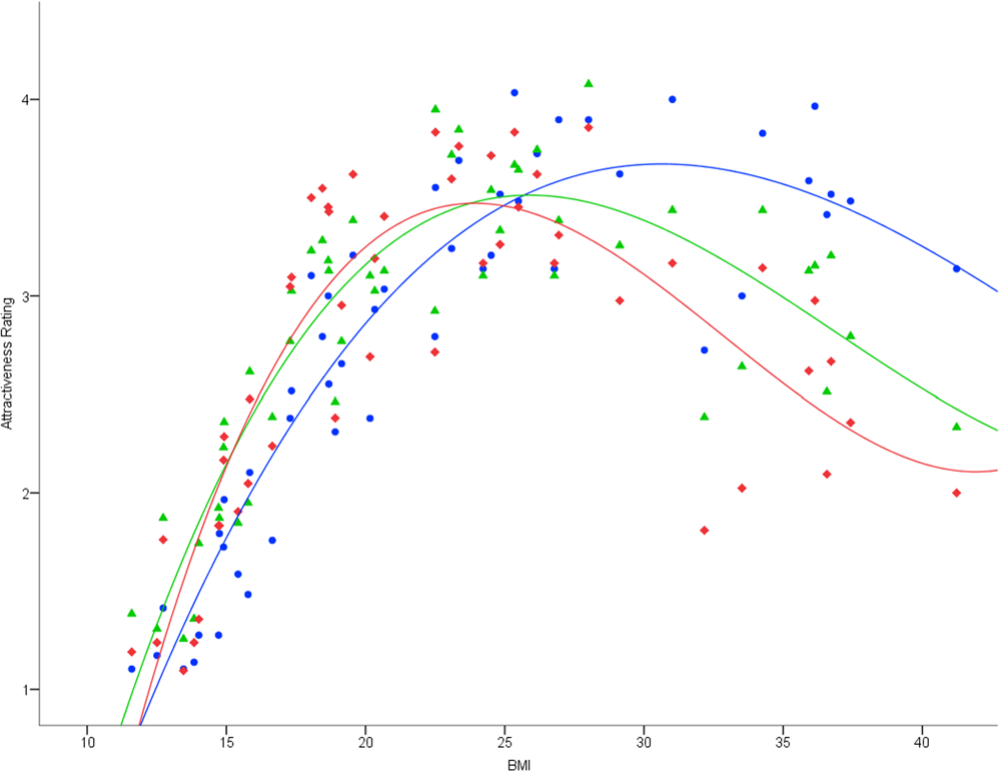
Cubic regression functions for the relationship between stimulus BMI and mean attractiveness rating by location (Village A: red line/lozenges; Village B: green line/triangles; Village C: blue line/circles).

ANCOVA was used to determine whether peak BMI preference differed between locations, with location and sex of participants entered as between-subjects variables, and age as covariate. There was a significant association between location and peak BMI preference (*F*_2, 103_ = 12.57, *p* < 0.001, ${\eta }_{p}^{2}$ = 0.19). Sidak-adjusted post hoc comparisons showed that residents of Village A had a lower peak BMI preference than residents of Village B (mean difference: −1.90, 95% CI [−3.78, −0.03], *p* = 0.045, *d* = 0.52), who in turn had a lower peak BMI preference than residents of Village C (mean difference: −2.23, 95% CI [−4.273, −0.18], *p* = 0.028, *d* = 0.58). There was also a significant association between sex and peak BMI preference (*F*_1, 103_ = 15.32, *p* < 0.001, ${\eta }_{p}^{2}$ = 0.13), so that male participants had a lower peak BMI preference than female participants (mean difference: −2.58, 95% CI [−3.89, −1.27], *p* < 0.001, *d* = 0.69). There was no interaction between sex and location (*F*_2, 103_ = 1.54, *p* = 0.219) and no main effect of age (*F*_1, 103_ = 1.11, *p* > 0.250). Cubic regression functions for the relationship between stimulus BMI and mean attractiveness rating by location are shown in Fig. 1.

### Predictors of BMI preference

Zero-order correlations showed 8 variables were significantly associated with peak BMI preference when considered in isolation, including TV consumption (*r* = −0.382, *p* < 0.001) and three of the nutritional variables (Diet quality: *r* = −0.189, *p* = 0.049; Food insecurity: *r* = −0.199, *p* = 0.037; Size of last meal: *r* = −0.216, *p* = 0.023; N for all analyses = 110; see full correlation matrix in Supplementary Table S2). Given the covariance of these variables across locations, however, Bayesian mixed effect multiple regression models were used to identify the most likely predictors of peak BMI preference. Given the high number of potential predictor variables in this study, Bayesian approaches allowed us to compare the likely probability of individual predictors driving peak BMI preference while increasing tolerance for power, and without enforcing one particular hierarchical structure between predictor variables on our data. That said, we also conducted frequentist analyses, which revealed very similar results (see Supplementary Analysis).

We employed a Bayesian mixed effects linear model using the STAN statistical package (Stan Development Team. 2016. Stan Modeling Language Users Guide and Reference Manual, Version 2.14.0. http://mc-stan.org). STAN performs Bayesian inference through Hamiltonian Monte Carlo sampling of a specified model. The model used includes hyper priors (priors over the parameters of the priors), which ensures that the data itself helps to constrain the priors over the effect sizes^28^. The code has been included in the Supplementary Note. For the sampling we used 4 traces, each with 10,000 samples after burn-in. To avoid auto-correlations we used every fifth sample leaving a total of 8,000 samples.

Since no interaction was found between sex and location for peak BMI preference (see previous section), men and women were analysed together. Location was entered as a random effect. In our first model, the 8 predictors which correlated significantly with peak BMI (see Supplementary Table S2) preference were entered as potential fixed effect predictors. Comparing the effect of the three locations showed that more than 97% of the probability mass of the estimated random effect of Location B and 99% for Location C were higher than for Location A, such that Location A still had lower body ideals despite inclusion of our predictors. However, effects of Location B and Location C did not meaningfully differ with a probability mass of 85% (i.e., the 8 variables accounted for all meaningful variation between these two locations).

**Figure 2:**
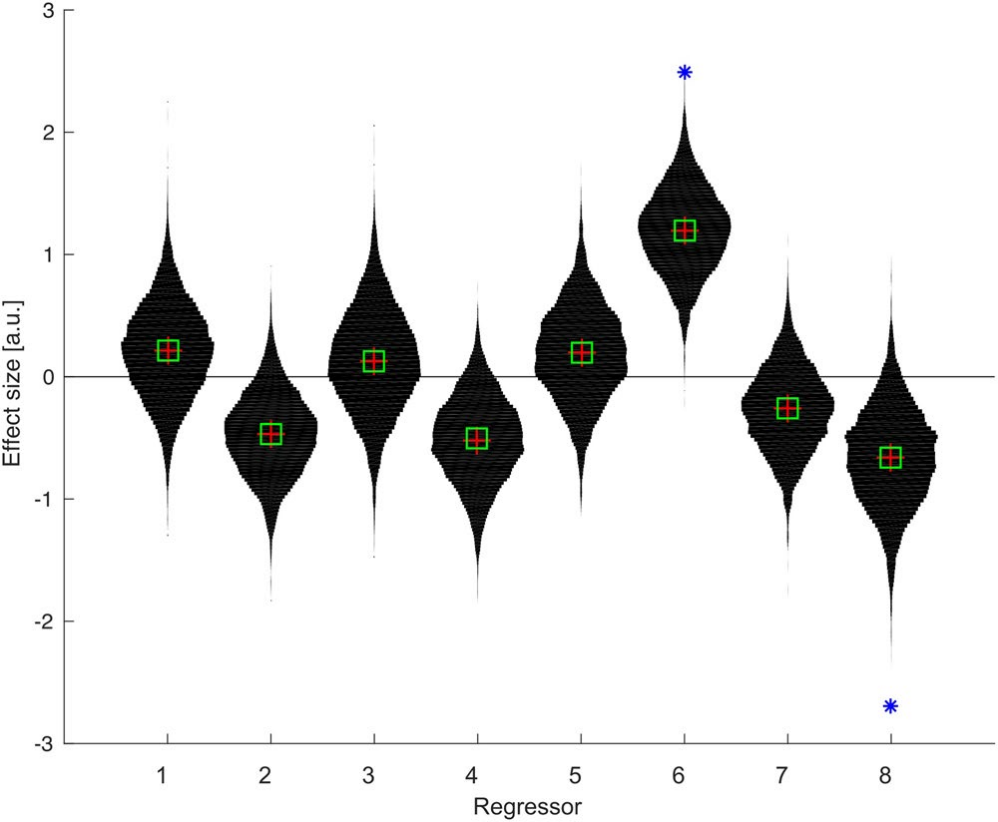
Violin plot of the 8 fixed effect regression coefficients (beta) of the mixed effects model where participants are clustered within villages. The red cross indicates the mean of each distribution, while the square is the median. Predictors: 1. Diet score, 2. Earnings, 3. Economic score, 4. Education (years), 5. Food insecurity, 6. Sex, 7. Size of last meal, 8. TV consumption (hours).

Considering the fixed effects, two regressors (TV consumption and Sex) had >95% probability mass away from the null line, implying a very likely effect of that regressor upon peak BMI preference (Fig. 2). Education and income both had probability masses over 90% away from the null, while the nutritional variables had only c. 63% and 70% mass away from the null – i.e. when considered alongside other predictors, they were unlikely to have a directional impact. Inclusion of all 14 potential independent variables, including those without significant associations with peak BMI preference, reduced the probability mass deviation of TV consumption to 90%; all other results remained qualitatively the same (see Table 3).

**Table 3:** Effect size and intercept estimates for both mixed effect linear models. Fixed effect estimates show un-signed percentage probability mass for effect size away from the null line for ease of comparison.

		Model 1	Model 2
Fixed effects	Diet quality	0.705	0.653
	Earnings	0.922	0.918
	Economic score	0.624	0.580
	Education	0.932	0.875
	Food insecurity	0.694	0.755
	Sex	0.999	0.998
	Size of last meal	0.785	0.777
	Television consumption	0.954	0.900
	Acculturation		0.733
	Age		0.630
	Hunger		0.834
	Time since last meal		0.715
	zBMI		0.643
	zWHR		0.704
Intercepts	Location A	25.687	25.518
	Location B	27.174	27.280
	Location C	28.207	28.322

See Fig. 2 for directional estimates.

## Discussion

The aim of the current study was to test the effect of television consumption on female body size ideals while controlling for a critical confounding variable: nutritional status or food insecurity. We compared female body size ideals in three Nicaraguan villages that represented different combinations of television access and nutritional status. Cluster analysis demonstrated that the villages differed both in terms of the quantity and the nutritional richness or variety of foods available to them.

Comparisons showed that both villages with high television access (Village A and Village B) preferred thinner female bodies than the village with very low television access (Village C). Additionally, in the two villages with high television access, the village with high nutritional status (Village A) preferred thinner bodies than the village with low nutritional status (Village B). Thus these results were superficially consistent with both television access and nutrition playing a role in determining female body size ideals.

However, frequentist and Bayesian regression models found no contribution of any of the nutritional variables to variance in female body size ideals. Instead, any differences between Village A and Village B not explained by television consumption seem to have been most likely due to other non-measured variables, as demonstrated by the strong likelihood found that the intercept for Village A was meaningfully different from Villages B and C. In contrast, television consumption was found to predict body ideals beyond these other variables, although inclusion of variables that were not initially associated with peak BMI preference weakened this result. The variables entered into the first model, however, were sufficient to account for the meaningful difference between Villages B and C, with television consumption (after sex, which was equally balanced across locations) the most likely predictor to explain variance in individuals’ body size preferences. As such we consider it highly likely that our two low-nutrition villages showed differences in body ideals which were most likely driven by TV consumption.

The fact that income was marginally more likely than TV to contribute to variation in Model 2 should be noted however; given the fact that earnings facilitate both TV consumption (via travel or paying for the TV/satellite TV subscription) we would certainly expect earnings to play a role. Indeed the full correlation table shows earnings correlate significantly with TV, nutrition, and body mass (Supplementary Table S2). Our previous work in this region, however, has noted a contribution of television consumption to female body size preferences that was independent of income^14^. Nevertheless, future studies with more power may wish to consider structural equation modelling to consider the likely causal relationships here. As to why the estimates for TV drop in the latter model despite the additional variables correlating with neither peak BMI preferences nor TV consumption in the zero-order correlations, we would suggest that our sample may partly lack power to detect small associations with so many variables contributing to even marginal amounts of variance.

The fact that the nutritional variables had a low likelihood of explaining variance in body size preferences in either model, and that neither model fully accounted for the difference between Village A (high media, high nutrition) and the low nutrition villages, leads us to conclude that we have no clear evidence for a role of long term nutrition in driving body ideals. Finally, as noted above, there was a strong association between participant sex and body size preference ideals, such that women preferred larger female figures, which is consistent with our previous observation that women are more tolerant than men of higher body weights in some rural communities in this region, even while the opposite pattern was found in the urban sample^14^.

Beyond any differences in television consumption, nutrition, and the socioeconomic factors we documented, non-measured factors that could have contributed to the observed difference between Village A (high TV, high nutrition) and Village B (high TV, low nutrition) include population size and density, and contact with outside cultural groups. When investigating facial attraction, Scott *et al*.^29^ found that population density was a significant predictor of masculinity preferences and seemed to be also associated with the strength of participants’ perceptions of an association between masculinity and negative personality traits. This would suggest that the greater density in Village A, and perhaps greater stratification due to engagement with the cash economy, may facilitate expression of evolutionarily novel preferences (for masculinity in Scott et al.’s data; for thinner bodies in ours). Furthermore, Village A has a small hotel and has more contact with tourists and individuals travelling from other locations in the lagoon region. This may facilitate greater general exposure to cultural concepts of industrialised populations (such as the thin ideal) even where media access is controlled, although we note that acculturation as measured in our data did not significantly differ between locations.

Another, less likely factor that could have contributed to the observed difference between Village A and Village B is health infrastructure. Although health infrastructure has also been shown to influence attractiveness ideals in some studies^30^ (but see too ref. 29), we believe that this is unlikely in our study site, because the three villages have a very similar access to health services. None of these villages has a hospital, and for acute health issues inhabitants of all three villages go to the same hospital in a larger nearby town. Additionally, medical brigades visit all the communities equally on government programmes for vaccination and other preventative treatments, and following long fieldwork in the area, we found no evidence than participants in Village A were healthier than participants in the other villages.

It should also be noted that none of the communities selected were starving or underweight at the time of data collection, so differences in the levels of nutritional status may have been insufficiently wide to find an effect of nutrition on female body size preference. However, the communities differed significantly on four of the five nutritional measures, and most importantly on food insecurity. Food insecurity measured participants’ seasonal risk of food scarcity, which, from an evolutionary point of view, should be the main determinant of female body size preference^7, 9^. In the current study, we had enough variation to test that hypothesis since the levels of food insecurity (and diet quality) clearly differed between communities. For example, out of the two communities with high television access, 49% of Village B participants reported that they experience periods of food scarcity during the year (item 6 of food insecurity questionnaire), whereas only 14% of Village A participants did. This, with the fact that participants’ BMI (and WHR) did not show a significant relationship with peak BMI preference, suggests that nutrition plays a minor role in determining female body size preference in the communities studied.

Another limitation concerns the stimuli used in the female figure preference task. The photographs used were of White European women, and perhaps the body size that our participants consider attractive in White women is not the same as the body size that they find attractive in women of their own ethnicity. In particular, participants may have different ideals when it comes to body shape or specific body parts^31^. Alternatively, the rating of White women could reflect an artificial association between ‘thinness’ and ‘white bodies’, without reflecting true preference for attractiveness. That said, in the current study, participants who have access to television watch programmes featuring predominantly Hispanic and White women (and not women of their own ethnicity). It therefore seemed appropriate to use stimuli depicting White women to achieve consistency between what participants see on the TV and the bodies they rated in this study. Further, previous research using the same set of bodies found that body size, not body shape, is the main determinant of physical attractiveness^27^, including in non-Western samples^11^. In other words, it is unlikely our participants used other considerations than weight when rating this specific set of bodies.

Despite the above limitations, our findings provide evidence that television consumption contributes more (albeit modestly) to determining female body size ideals in previously media-naive populations than virtually all other potential influencing factors. In this study, television consumption was not only a more likely predictor of BMI preference than nutrition, but also than acculturation, age, several measures of socio-economic status, and even participant BMI. Notably, any effect of television in these results arises from relatively recent and moderate television exposure. The average participant tested in Village B was not exposed to television until of the age of 28 years old (given that electricity was gradually introduced from 2009, and that the average age of participants tested was 34 years old in 2015), and the average television consumption across Village A and Village B was less than 14 hours per week. This contrasts sharply with the age at which most Westerners are first exposed to the thin-body ideal, and the omnipresence of the latter in the Western media (not only on television, but also in magazines and on the internet, to which the communities tested have almost no access). However, we found that such a moderate media exposure likely had an effect on participants’ female body size ideals (in Villages A and B in particular), and accounted for variation between communities better than any other measured factor which varied across locations.

While previous research has shown that media exposure can significantly impact body ideals, the current study found that even in the face of constraints as basic as poor nutritional status, television consumption may still be implicated in driving the preference for a lower weight female body. This is an important finding if one considers that the thin-body ideal can negatively impact body satisfaction and thereby be a major factor in the development of national-scale trends in psychopathologies, including in non-Western populations.

## Electronic supplementary material

Supplementary Information
