# Supplementary material for: Nutritional status and the influence of TV consumption on female body size ideals in populations recently exposed to the media
Source: Sci Rep. 2017 Aug 16;7:8438. doi: 10.1038/s41598-017-08653-z (PMC5559456; doi:10.1038/s41598-017-08653-z)
Supplement: Supplementary file 1 — Supplementary Information [file 41598_2017_8653_MOESM1_ESM.pdf]

Supplementary Information

Article: Nutritional status and the influence of TV consumption on female body size ideals in populations recently exposed to the media

Authors: Jean-Luc Jucker, Tracey Thornborrow, Ulrik Beierholm, D. Michael Burt, Robert A. Barton, Elizabeth H. Evans, Mark A. Jamieson, Martin J. Tovee, and Lynda G. Boothroyd

Contents:

Supplementary Methods. Food insecurity questionnaire .... p. 2

Supplementary Table S1. Two-step cluster analysis of nutrition data.............................. p. 3

Supplementary Note. Bayesian analysis: Stan Model code.. p. 4

Supplementary Analysis. Frequentist analyses .... p. 7

Supplementary Table S2. Full correlation matrix.. p. 9

Supplementary Table S3. Hierarchical regression analyses .... p. 10

Supplementary Methods. Food insecurity questionnaire

1. How many meals do you have in a typical day? (*three or more, two or less*)
2. Do you have enough food to eat in a typical day? (*yes, no*)
3. Do all members of your household have enough food to eat in a typical day? (*yes, no*)
4. Where does most of the food you consume come from? (*mainly from shops, mainly from fishing or farming*)
5. Are there periods in the year when you diet changes significantly? (*yes, no*)
   - If so, specify period and diet (open-ended)
6. Are there periods in the year when it is more difficult to find food (e.g., crops or fish) or during which you are hungrier? (*yes, no*)
   - If so, specify period (open-ended)
7. Can you choose what you want to eat every day? (*yes, no*)
8. Do you sometimes wish you could eat something different or do you sometimes miss some foods (e.g., meat)? (*yes, no*)
9. In comparison with the surrounding communities, do you consider that your community has easier access or more difficult access to food and varied foods? (easier, more difficult)

Note. Answers to items 1-9 were coded as 0 and 1 and were summed for each participant, with a high score indicating a high food insecurity. Items 5, 6, and 8 were reversed when coding the data. Open-ended answers are not discussed in the current study.

Supplementary Table S1. Two-step cluster analysis of nutrition data

	Predictor importance	Cluster 1	Cluster 2
Beans	0.52	5.89	3.89
Bread	0.78	6.36	3.02
Breadkind (e.g., cassava)	0.25	6.63	6.99
Cheese	1.00	2.47	0.32
Eggs	0.03	3.22	2.90
Fish and seafood	0.01	5.72	5.80
Fowl meat and red meat	0.89	1.92	0.69
Fruits	0.45	3.59	1.90
Oil	0.49	6.18	4.59
Processed foods	0.47	2.38	1.47
Rice	0.28	6.90	6.07
Sugared beverages	0.25	4.74	4.11
Vegetables	0.69	2.76	1.09

Note. Some items were grouped for analysis. For example, coffee/tea with sugar, soft drinks, and sugared squash were grouped as ‘sugared beverages’.

Supplementary Note. Bayesian analysis**:** Stan Model code

```
data {
      int<lower=0> N1;  // number of data items
      int<lower=0> N2;  // number of data items
      int<lower=0> N3;  // number of data items
      int<lower=0> K;  // number of predictors

      matrix[N1, K] x1;  // predictor matrix
      vector[N1] y1;     // outcome vector
      matrix[N2, K] x2;  // predictor matrix
      vector[N2] y2;     // outcome vector
      matrix[N3, K] x3;  // predictor matrix
      vector[N3] y3;     // outcome vector
}

parameters {
   //real beta0;            // intercept
   real beta01;            // intercept
   real beta02;            // intercept
   real beta03;            // intercept

vector[K] beta1;                    // coefficients for predictors
vector[K] beta2;                    // coefficients for predictors
vector[K] beta3;                    // coefficients for predictors

         real<lower=0> sigma;           //error scale

         vector[K]    betamu;            //beta prior
   real<lower=0>    betasigma;          //beta prior

   //real    betamu2;            //beta prior
   //real<lower=0>    betasigma2;         //beta prior

   //real    betamu3;            //beta prior
   //real<lower=0>    betasigma3;         //beta prior
```

```
    //real    betahmu;              //beta hyper prior
    //real<lower=0>    betahsigma;           //beta hyper prior
}

model {
      y1 ~ normal(x1 * beta1 + beta01, sigma);  // likelihood
      //beta1 ~ normal(betamu1,betasigma1);      // specify prior?
      y2 ~ normal(x2 * beta2 + beta02, sigma);  // likelihood
      //beta2 ~ normal(betamu2,betasigma2);      // specify prior?
      y3 ~ normal(x3 * beta3 + beta03, sigma);  // likelihood
      //beta3 ~ normal(betamu3,betasigma3);      // specify prior?

      for (k in 1:K){
            beta1[k]~normal(betamu[k],betasigma);
            beta2[k]~normal(betamu[k],betasigma);
            beta3[k]~normal(betamu[k],betasigma);}

      beta01 ~ normal(0,50);                // specify prior?
      beta02 ~ normal(0,50);                // specify prior?
      beta03 ~ normal(0,50);                // specify prior?
      sigma ~ gamma(7, 1);                  // specify prior?

      betamu ~ normal(0,10);
      betasigma ~ gamma(2,1);//7,1);

      //betamu2 ~ normal(betahmu,10);
      //betasigma2 ~ gamma(betahsigma,1);

      //betamu3 ~ normal(betahmu,10);
      //betasigma3 ~ gamma(betahsigma,1);

      //betahmu ~ normal(0,10);
      //betahsigma ~ gamma(7,1);


}
generated quantities {
```

```
real ll1 ;
vector[N1+N2+N3] ll3 ;

ll1<-normal_log(y1 , x1 * beta1 + beta01, sigma)+normal_log(y2 , x2 * beta2 + beta02,
sigma)+normal_log(y3 , x3 * beta3 + beta03, sigma);

for (n in 1:N1)
   ll3[n]<-normal_log(y1[n] , x1[n] * beta1 + beta01, sigma);
for (n in 1:N2)
   ll3[n+N1]<-normal_log(y2[n] , x2[n] * beta2 + beta02, sigma);
for (n in 1:N3)
   ll3[n+N1+N2]<-normal_log(y3[n] , x3[n] * beta3 + beta03, sigma);
}
```

Supplementary Analysis. Frequentist Analyses

Hierarchical regression models were used to identify predictors of peak BMI preference. Out of the fourteen independent variables, eight were found to significantly correlate with peak BMI preference and were therefore considered as potential predictors (full correlation matrix is shown in Supplementary Table S2; the variables BMI and WHR were standardised as they had been found to differ between sex). They were television consumption, three measures of nutritional status (diet quality score, food insecurity score, and size of last meal), as well as four control variables (earnings, economic score, education, and sex). Since no interaction was found between sex and location for peak BMI preference (see Results section), men and women were analysed together. All model coefficients are shown in Supplementary Table S3.

There were no multicollinearity issues as none of the predictors used in regression analyses had intercorrelations higher than 0.5, and tolerance values were higher than 0.6 across all analyses. Further, across all analyses, there were no studentized deleted residuals higher than ±3 standard deviations, and although a few leverage values were higher than 0.2 (up to 0.38 for one observation), there were no values for Cook's distance above 1 across all analyses (the observation with a 0.38 leverage had a corresponding Cook's value of 0.15, showing that it had a relatively low influence, and was therefore not discarded from analyses). Finally, across all analyses the residuals were approximately normally distributed as assessed by Q-Q plots.

To start with, all participants were analysed together and the four control variables were entered in a first model. Either nutritional status (second model) or television (third model) were then added to this initial model. When nutritional status was added, the initial model did not improve (R^2 change = 0.034, $F_{3, 90}$ = 1.42, p = .241) and none of the nutritional measures predicted peak BMI preference. In contrast, when television consumption was added, the initial model improved (R^2 change = 0.068, $F_{1, 92}$ = 9.18, p = .003, f^2 = 0.272), and the only significant predictors were sex and television consumption, such that a lower peak BMI preference was associated with male gender and more TV consumption.

Comparisons between locations (see previous section) had shown that Village B and Village C differed on peak BMI preference and on television consumption, but not on nutritional status, suggesting that television consumption is the main determinant of female body size preferences. In contrast, Village A and Village B differed on peak BMI preference and on nutritional status, but not on television consumption, suggesting that nutritional status better accounts for female body size preference.

To clarify these results, separate regressions were run for Village B and Village C data together, and then for Village A and Village B data together. (We did not run

regressions for Village A and Village C data together because these communities differed on both television consumption and nutritional status). Using the same variables and the same regression method as above, adding nutritional status did not improve the initial models (Village B and Village C: R^2 change = 0.028, $F_{3, 57}$ = 0.77, p > .250; Village A and Village B: R^2 change = 0.025, $F_{3, 62}$ = 0.67, p > .250), whereas adding television consumption resulted in a significant improvement (Village B and Village C: R^2 change = 0.053, $F_{1, 59}$ = 4.70, p = .034, f^2 = 0.188; Village A and Village B: R^2 change = 0.055, $F_{1, 64}$ = 4.72, p = .033, f^2 = 0.280), leaving again sex and television consumption as the only significant predictors of peak BMI preference in the final models.

Regressions were finally used to rule out the possibility that the differences in peak BMI preference between the above locations could be due to other unmeasured variables. To do so, all variables used above were entered together in a first model, to which location was added hierarchically. Location did not improve the first model for either Village B and Village C (R^2 change = 0.004, $F_{1, 55}$ = 0.35, p > .250) or Village A and Village B (increase in R^2 change = 0.013, $F_{1, 60}$ = 1.055, p > .250.

Supplementary Table S2. Full correlation matrix (N for all analyses = 110; *p < .05, **p < .01)

		Peak BMI preference	Accultu-ration	Age	Diet quality	Earnings	Economic score	Education	Food insecurity	Hunger	Sex	Size of last meal	Television consumption	Time since last meal	zBMI	zWHR
Peak BMI preference	*r*		-.151	.099	-.189*	-.317**	-.268**	-.255**	.199*	.073	.295**	-.216*	-.382**	-.116	-.123	.072
	p		.120	.304	.049	.001	.005	.007	.037	.451	.002	.023	.000	.226	.210	.461
Acculturation	*r*	-.151		-.102	-.013	.330**	.023	.262**	.157	-.006	.063	.068	.085	.039	.225*	-.116
	p	.120		.294	.892	.001	.810	.007	.107	.949	.522	.487	.383	.690	.022	.244
Age	*r*	.099	-.102		-.203*	.061	-.148	-.247**	.034	-.171	-.083	-.148	-.158	.117	.219*	.428**
	p	.304	.294		.033	.549	.122	.009	.722	.075	.391	.123	.099	.223	.024	.000
Diet quality	*r*	-.189*	-.013	-.203*		.242*	.483**	.251**	-.512**	.138	-.033	.130	.350**	-.071	-.042	-.011
	p	.049	.892	.033		.016	.000	.008	.000	.149	.728	.176	.000	.460	.669	.913
Earnings	*r*	-.317**	.330**	.061	.242*		.286**	.209*	-.191	.053	-.143	.091	.293**	-.080	.337**	.215*
	p	.001	.001	.549	.016		.004	.039	.060	.606	.160	.375	.003	.436	.001	.037
Economic score	*r*	-.268**	.023	-.148	.483**	.286**		.341**	-.355**	.121	-.071	.007	.398**	-.048	.143	.056
	p	.005	.810	.122	.000	.004		.000	.000	.208	.458	.945	.000	.615	.144	.569
Education	*r*	-.255**	.262**	-.247**	.251**	.209*	.341**		-.088	.196*	.183	.134	.390**	-.026	.131	-.125
	p	.007	.007	.009	.008	.039	.000		.359	.040	.056	.163	.000	.784	.180	.200
Food insecurity	*r*	.199*	.157	.034	-.512**	-.191	-.355**	-.088		-.269**	-.032	-.241*	-.287**	.094	-.046	-.131
	p	.037	.107	.722	.000	.060	.000	.359		.005	.742	.011	.002	.327	.641	.180
Hunger	*r*	.073	-.006	-.171	.138	.053	.121	.196*	-.269**		.285**	.223*	.082	-.523**	-.009	.108
	p	.451	.949	.075	.149	.606	.208	.040	.005		.003	.019	.393	.000	.929	.269
Sex	*r*	.295**	.063	-.083	-.033	-.143	-.071	.183	-.032	.285**		.051	.090	-.112	-.009	.025
	p	.002	.522	.391	.728	.160	.458	.056	.742	.003		.594	.348	.242	.929	.798
Size of last meal	*r*	-.216*	.068	-.148	.130	.091	.007	.134	-.241*	.223*	.051		.280**	.115	.082	.090
	p	.023	.487	.123	.176	.375	.945	.163	.011	.019	.594		.003	.230	.403	.360
Television consumption	*r*	-.382**	.085	-.158	.350**	.293**	.398**	.390**	-.287**	.082	.090	.280**		.048	.109	-.123
	p	.000	.383	.099	.000	.003	.000	.000	.002	.393	.348	.003		.621	.267	.208
Time since last meal	*r*	-.116	.039	.117	-.071	-.080	-.048	-.026	.094	-.523**	-.112	.115	.048		.069	.102
	p	.226	.690	.223	.460	.436	.615	.784	.327	.000	.242	.230	.621		.485	.300
zBMI	*r*	-.123	.225*	.219*	-.042	.337**	.143	.131	-.046	-.009	-.009	.082	.109	.069		.304**
	p	.210	.022	.024	.669	.001	.144	.180	.641	.929	.929	.403	.267	.485		.002
zWHR	*r*	.072	-.116	.428**	-.011	.215*	.056	-.125	-.131	.108	.025	.090	-.123	.102	.304**	
	p	.461	.244	.000	.913	.037	.569	.200	.180	.269	.798	.360	.208	.300	.002	

Supplementary Table S3. Hierarchical regression analyses of predictors of peak BMI preference

			B (95% CI)	*β*	*t*	*p*
All participants	First model[1]	Earnings	-.001 (-.001, -.001)	-.185	-1.925	.057
		Economic score	-.093 (-.235, .048)	-.129	-1.311	.193
		Education	-.281 (-.504, -.058)	-.246	-2.505	.014
		Sex	2.487 (1.003, 3.972)	.309	3.328	.001
	Second model[2]	Earnings	-.001 (-.001, .000)	-.163	-1.692	.094
		Economic score	-.085 (-.240, .071)	-.117	-1.082	.282
		Education	-.262 (-.488, -.036)	-.230	-2.306	.023
		Sex	2.514 (1.020, 4.009)	.312	3.343	.001
		Diet quality	-.005 (-.075, .065)	-.016	-.138	.890
		Food insecurity	.127 (-.439, .693)	.049	.446	.657
		Size of last meal	-1.221 (-2.610, .167)	-.164	-1.748	.084
	Third model[3]	Earnings	.000 (-.001, .000)	-.130	-1.390	.168
		Economic score	-.040 (-.180, .101)	-.055	-.561	.576
		Education	-.188 (-.411, .034)	-.165	-1.682	.096
		Sex	2.695 (1.265, 4.125)	.335	3.744	.000
		TV consumption	-.152 (-.252, -.052)	-.304	-3.031	.003
Village B & Village C	First model[4]	Earnings	-.001 (-.002, .000)	-.222	-1.835	.071
		Economic score	-.054 (-.248, .140)	-.066	-.555	.581
		Education	-.182 (-.443, .080)	-.165	-1.388	.170
		Sex	3.089 (1.305, 4.873)	.384	3.464	.001
	Second model[5]	Earnings	-.001 (-.002, .000)	-.221	-1.792	.078
		Economic score	-.097 (-.305, .110)	-.118	-.938	.352
		Education	-.189 (-.454, .076)	-.172	-1.427	.159
		Sex	3.191 (1.334, 5.047)	.396	3.442	.001
		Diet quality	.027 (-.066, .120)	.079	.581	.563
		Food insecurity	-.085 (-.814, .645)	-.030	-.232	.817
		Size of last meal	-1.071 (-2.663, .521)	-.158	-1.347	.183
	Third model[6]	Earnings	-.001 (-.001, .000)	-.163	-1.353	.181
		Economic score	-.004 (-.198, .190)	-.005	-.042	.967
		Education	-.145 (-.401, .111)	-.132	-1.132	.262
		Sex	3.308 (1.565, 5.052)	.411	3.797	.000
		TV consumption	-.136 (-.262, -.010)	-.258	-2.168	.034
Village A & Village B	First model[7]	Earnings	.000 (-.001, .000)	-.132	-1.151	.254
		Economic score	-.093 (-.268, .083)	-.122	-1.053	.296
		Education	-.274 (-.574, .026)	-.213	-1.823	.073
		Sex	2.626 (.820, 4.431)	.335	2.905	.005

Second model[8]	Earnings	.000 (-.001, .000)	-.115	-.979	.331
	Economic score	-.061 (-.252, .130)	-.080	-.634	.528
	Education	-.229 (-.541, .083)	-.178	-1.466	.148
	Sex	2.559 (.695, 4.424)	.327	2.744	.008
	Diet quality	-.029 (-.117, .059)	-.092	-.663	.509
	Food insecurity	.047 (-.620, .715)	.020	.142	.888
	Size of last meal	-.847 (-2.702, 1.009)	-.111	-.912	.365
Third model[9]	Earnings	.000 (-.001, .000)	-.101	-.899	.372
	Economic score	-.080 (-.251, .091)	-.105	-.931	.355
	Education	-.158 (-.468, .153)	-.123	-1.012	.315
	Sex	2.895 (1.121, 4.669)	.370	3.261	.002
	TV consumption	-.141 (-.270, -.011)	-.261	-2.173	.033

1. R^2 = .250, F[4, 93] = 7.758, p < .0001; 2. R^2 = .284, F[7, 90] = 5.103, p < .0001; 3. R^2 = .318, F[5, 92] = 8.590, p < .0001; 4. R^2 = .281, F[4, 60] = 5.874, p < .0001; 5. R^2 = .309, F[7, 57] = 3.649, p < .005; 6. R^2 = .334, F[5, 59] = 5.929, p < .0001; 7. R^2 = .196, F[4, 65] = 3.962, p < .01; 8. R^2 = .221, F[7, 62] = 2.518, p < .05; 9. R^2 = .251, F[5, 64] = 4.296, p < .005.
